# O.3.3-2 Promoting physical activity to patients: a scoping review of the perceptions of doctors in the United Kingdom

**DOI:** 10.1093/eurpub/ckad133.157

**Published:** 2023-09-11

**Authors:** Graham Baker, Gemma Woodhead, Divya Sivaramakrishnan

**Affiliations:** University of Edinburgh, UK; graham.baker@ed.ac.uk; University of Edinburgh, UK; graham.baker@ed.ac.uk; University of Edinburgh, UK; graham.baker@ed.ac.uk

## Abstract

**Purpose:**

The physician–patient encounter presents an ideal opportunity for physical activity (PA) promotion. This review aims to: i) explore the breadth and depth of existing literature investigating doctors’ perceptions of PA promotion in the United Kingdom (UK); and ii) identify factors influencing the extent to which doctors engage in PA promotion during patient interactions.

**Methods:**

A five-stage scoping review methodology and the PRISMA-ScR guidance were followed. Stage 1 – research questions specified. Stage 2 – relevant studies identified by searching five electronic databases and manual screening of references. Stage 3 – studies screened using Covidence™. Stage 4 – study data extracted and charted. Stage 5 – findings from included studies were analysed, summarised and reported using: i) descriptive numerical analysis to provide insight into study characteristics; and ii) narrative summary of the evidence categorised by factors that influence doctors’ engagement with PA promotion.

**Results:**

In total, 16,961 studies were screened. Nineteen studies were included in the review with most conducted in primary care focusing on the perceptions of general practitioners. Seven influencing factors were identified: knowledge and training, personal interest and PA level, time, resources, confidence, the perceived role of the doctor and patient relevance.

**Conclusions:**

This review provides new evidence that historical barriers and influencing factors have a persisting impact on the ability and willingness of UK doctors to engage with PA promotion with patients. Previous efforts to address these factors would appear to have had limited success. Further intervention efforts are required to ensure more widespread and effective PA promotion to patients.

